# The role of posttranslational modifications in generating neo-epitopes that bind to rheumatoid arthritis-associated HLA-DR alleles and promote autoimmune T cell responses

**DOI:** 10.1371/journal.pone.0245541

**Published:** 2021-01-19

**Authors:** Stephane Becart, Karen B. Whittington, Amanda Prislovsky, Navin L. Rao, Edward F. Rosloniec

**Affiliations:** 1 Immunology Discovery, Janssen Research and Development LLC, San Diego, California, United States of America; 2 Veterans Affairs Medical Center, Memphis, Tennessee, United States of America; 3 Immunology Discovery, Janssen Research and Development LLC, Spring House, Pennsylvania, United States of America; 4 Department of Medicine, University of Tennessee Health Science Center, Memphis, Tennessee, United States of America; 5 Department of Pathology, University of Tennessee Health Science Center, Memphis, Tennessee, United States of America; Duke University School of Medicine, UNITED STATES

## Abstract

While antibodies to citrullinated proteins have become a diagnostic hallmark in rheumatoid arthritis (RA), we still do not understand how the autoimmune T cell response is influenced by these citrullinated proteins. To investigate the role of citrullinated antigens in HLA-DR1- and DR4-restricted T cell responses, we utilized mouse models that express these MHC-II alleles to determine the relationship between citrullinated peptide affinity for these DR molecules and the ability of these peptides to induce a T cell response. Using a set of peptides from proteins thought to be targeted by the autoimmune T cell responses in RA, aggrecan, vimentin, fibrinogen, and type II collagen, we found that while citrullination can enhance the binding affinity for these DR alleles, it does not always do so, even when in the critical P4 position. Moreover, if peptide citrullination does enhance HLA-DR binding affinity, it does not necessarily predict the generation of a T cell response. Conversely, citrullinated peptides can stimulate T cells without changing the peptide binding affinity for HLA-DR1 or DR4. Furthermore, citrullination of an autoantigen, type II collagen, which enhances binding affinity to HLA-DR1 did not enhance the severity of autoimmune arthritis in HLA-DR1 transgenic mice. Additional analysis of clonal T cell populations stimulated by these peptides indicated cross recognition of citrullinated and wild type peptides can occur in some instances, while in others cases the citrullination generates a novel T cell epitope. Finally, cytokine profiles of the wild type and citrullinated peptide stimulated T cells unveiled a significant disconnect between proliferation and cytokine production. Altogether, these data demonstrate the lack of support for a simplified model with universal correlation between affinity for HLA-DR alleles, immunogenicity and arthritogenicity of citrullinated peptides. Additionally they highlight the complexity of both T cell receptor recognition of citrulline as well as its potential conformational effects on the peptide:HLA-DR complex as recognized by a self-reactive cell receptor.

## Introduction

The association between the expression of specific MHC class II (MHC-II) alleles and susceptibility to autoimmunity has been recognized for many years [1–3]; yet, we still do not fully understand how a particular HLA allele predisposes an individual to developing an autoimmune disease. While implicit in these associations is a role for CD4+ T cells, how these MHC class II alleles selectively stimulate self-reactive CD4+ T cells remains unknown. MHC-II molecules have two primary functions—binding peptides, and in complex with the peptides, serving as ligands for T cell receptors (TCR) on CD4+ T cells. Peptide binding is dependent on the interaction of amino acid side chains of the peptide with the P1, P4, P6, and P9 pockets located in the floor and walls of the MHC-II binding groove [4]. MHCII allele polymorphisms are concentrated in these binding pockets, thus varying peptide binding specificity among alleles and the likely basis for autoimmune susceptibility. How this differs from the response to foreign antigens and translates into the stimulation of pathogenic T cells specific for self-peptides is unclear.

Rheumatoid arthritis (RA) is an autoimmune disease in which susceptibility is associated with expression of several HLA-DR alleles, including DR1 (DRB1*01:01) and DR4 (DRB1*04:01, *04:04, *04:05, and *04:08) [5–7]. The commonality among the DR alleles associated with RA susceptibility is a stretch of amino acids at positions 70 thru 74 (Q/R-K/R-R-A-A) in the DRB1 chains termed the shared epitope (SE) [8–10]. This amino acid sequence motif is found in all RA-associated DRB1 alleles [2, 8, 11] and the SE-coding HLA-DRB1 alleles not only confer a higher risk for RA [12] but also earlier disease onset [13]. Furthermore, there is evidence of a gene-dose effect, in which the risk in developing RA correlates positively with the number of SE-coding HLA-DRB1 alleles [14–16]. Finally, the shared epitope has also been shown to positively associate with disease severity and bone erosion [17, 18]. The SE polymorphism is located within the P4 binding pocket of the HLA-DR molecule, implying that the SE plays a role in the allelic specificity of peptide binding by RA- associated DR alleles. Crystal structures of RA-associated DRB1 molecules indicate that side chains of SE amino acids interact with a bound peptide while others have the potential to contact the TCR [19–21]. Genetic analyses of DR alleles expressed by RA patients strongly implicate SE amino acids 70 and 71 in the risk for disease development [2].

While the SE polymorphisms are the most significant genetic factor associated with susceptibility to RA, the autoantigens that are involved in the pathogenic T cell response are elusive. In recent years, several clinical observations have implicated proteins that have undergone posttranslational modifications (PTM) as potential antigenic targets of the autoimmune response in RA [22]. PTM are biological processes that alter amino acid side chains after protein synthesis. In RA, the PTM of arginine (Arg) to citrulline (Cit) has become a major focus of study and the presence of antibodies to Cit proteins (called Anti-Citrullinated Protein Antibodies or ACPA) has become a diagnostic hallmark of this autoimmune disease [23, 24]. ACPA are highly specific to the disease [13, 25] and may directly influence joint inflammation and erosion through local binding to citrullinated proteins [23, 26, 27]. Genetic analyses of RA patients have indicated the SE is tightly linked to the presence of anti-Cit antibodies [28], suggesting that the SE alleles are directly involved in the development of the anti-Cit response. Several proteins including vimentin, type II collagen, and fibrinogen have been suggested to be targets of citrullination [29–33], and the Cit-peptides derived from these proteins targets of the autoimmune T cell response in RA [34].

Citrullination could confer immunogenicity to a self-peptide in two ways. First, citrullination of a side chain could generate a peptide that now has the capacity to bind to the class II allele, thus generating a novel MHC-peptide complex for T cell stimulation [21, 35]. Second, the PTM may alter a TCR contact residue on a peptide that already has the capacity to bind to the class II allele. Several studies investigating the role of Cit-proteins in the stimulation of autoimmune T cells have focused on the accommodation of Cit side chains in the binding pockets of DR molecules and its effect on peptide binding affinity. Studies by Hill *et al*. have demonstrated that a vimentin peptide with a Cit residue at the P4 location bound with a higher affinity to DR4 than the WT peptide [36], suggesting that Cit peptides have an enhanced affinity for DR alleles. These studies were extended by James *et al*. [37], demonstrating that the binding pockets in the DR1 allele, especially P1 and P4, will accept Cit side chains. While these studies demonstrated the ability of Cit side chains to be accommodated by the DR binding pockets of some alleles, the amino acid positions changed to Cit in these studies were selected on the basis that the Cit residues would only be in binding pockets. Recent studies by Sidney *et al*., have demonstrated that citrullination infrequently generates a peptide that has enhanced binding to HLA-DR, and the authors suggest that Cit more likely influences TCR contact with the Cit-peptide:DR complex than peptide binding [35]. While collectively these data indicate a potential role of citrullinated autoantigens in autoimmunity, questions remain as to the role of Cit in the stimulation of T cell responses. In the studies described here, we examined the role of citrullinated antigens in the generation of T cell responses restricted to HLA-DR1 and DR4. Using DRB1*01:01 and DRB1*04:01 humanized mouse models, we assessed the relationship between HLA-DR binding affinity and T cell immunogenicity of WT and Cit forms of vimentin, aggrecan, type II collagen, and fibrinogen peptides and proteins associated with the autoimmune T cell response in RA [20, 21, 35, 36, 38]. Our results indicate a complex array of scenarios. While citrullination of a peptide can enhance its ability to bind to the DRB1*01:01 or DRB1*04:01 alleles, this is not true for all citrullinated peptides. Additionally, enhanced binding affinity of the Cit-peptides to these HLA-DR alleles was not a reliable predictor for T cell immunogenicity. In instances where Cit-specific T cell responses were generated, some were found to also recognize the WT version of the antigenic peptide at the clonal level, while others were clearly novel and consistent with the concept of epitope spreading. In all, these data indicate that while citrullination of proteins can potentially generate novel T cell determinants, it is not a universal phenomenon, and its effect on T cell stimulation can be the result of the generation of a peptide that either now binds or binds with a higher affinity to the HLA-DR molecule, or the citrulline serves as a ligand or the TCR.

## Materials and methods

### Mice

The generation of DRB1*0101 and DRB1*04:01 transgenic mice (DR1) has been previously described [39, 40]. The DRA1 and DRB1 transgenes were established in (C57BL/6 x SJL/J) F2 mice, backcrossed to the B6 background, and the I-Ab molecule expressed by the B6 mice was genetically deleted by backcrossing the B6.129-H2^dlAb1-Ea^ knockout (stock number 003584, Jackson Laboratories). All mice used in these studies were bred in our facility and maintained in micro-isolators in a pathogen-free environment and were fed standard rodent chow (Ralston Purina) and water ad libitum. The animal portion of these studies was approved by the IACUC at the Memphis VA Medical Center, approval #943218. Euthanasia was performed by cervical dislocation under isofluorane anesthesia.

### Synthetic peptides

Wild type (WT) and citrullinated synthetic peptides of vimentin (Vim), type II collagen (CII), aggrecan (Agg), and fibrinogen (Fib) were used in these experiments (Table 1) (RS synthesis, Louisville, Ky). The vimentin (Vim) and citrullinated vimentin (Cit-Vim) peptides were dissolved in PBS containing 0.05% DMSO and used at 1 mg/ml for immunization. For proliferation assays, the Vim peptides were dissolved in HL-1 + 0.05% DMSO (1 mg/ml). DMSO was added first to the peptide, and then the PBS or HL-1 was added while vortexing (1 μl DMSO/2 ml of PBS or HL-1).

**Table 1:** Sequences of peptides tested for their ability to bind to HLA-DR1 and DR4 and to induce a CD4+ T cell response in mice expressing these HLA-DR allotypes.

Peptide	Sequence
Aggrecan (84–103; R-93-Cit)	VVLLVATEG**R**VRVNSAYQDK
Type II collagen (1236–1249; R-1240-Cit)	LQYM**R**ADQAAGGLR
Fibrinogen-α (78–91; R-84-Cit)	NQDFTN**R**INKLKNS
Vimentin (66–78; R-71-Cit)	SAVRL**R**SSVPGVR

Underlined residues are putative binding anchors P1, P4, P6, and P9 for the peptides, in respective order. P7 was omitted because of peptide variability at this position and lack of structural data to support it functioning as an anchor for all peptides. **R** indicates arginine residues that were substituted with citrulline.

The collagen (CII) peptides, CII(1236–1249) WT, Cit-CII(1236–1249), and the CII(257–274) WT were dissolved at 1 mg/ml in 10 mM acetic acid. PBS was used as the diluent buffer for immunization, and HL-1 for proliferation assays. Maximum of 20 μg of peptide was used per well in proliferation assays, keeping the acetic acid concentration in culture at 0.6 mM at its highest concentration. Previous studies using acetic acid solubilized peptides in proliferation assays have indicated that this concentration does not alter the T cell response.

Aggrecan (Agg) and citrullinated Agg (Cit-Agg) peptides were dissolved at 1 mg/ml in 10 mM acetic acid for immunization (PBS) and proliferation assays (HL-1). Maximum of 20 μg of peptide was used per well in proliferation assays as discussed for the CII peptides.

The fibrinogen (Fib) peptide was dissolved in PBS (1 mg/ml, for immunization) and in HL-1 (1 mg/ml, for proliferation). The citrullinated fibrinogen peptide (R84Cit; Cit-fib) was solubilized at 42°C in 0.5% DMSO in PBS or HL-1 at 1 mg/ml. The highest final concentration of DMSO at 0.166% in the proliferation assays (antigen peptide diluted 1:3) was not inhibitory to T cell proliferation.

### Citrullination of CII

Citrullinated CII (Cit-CII) was produced using the method described by Lundberg *et al*. [23] and Burkhardt *et al*. [41]. 4 ml of CII at 0.25 mg/ml was dialyzed against citrullination buffer (0.1 M Tris-HCl pH 7.6, 10 mM CaCl_2_, 5 mM DTT), and 6.7 μl (8.5 μg, 33.4 U) of PAD4 (human recombinant, Cayman Chemical) was used to induce citrullination. Sham Cit-CII was produced using the same procedure without the PAD4 enzyme.

To assess the structural integrity and recovery of the CII molecules, all samples were analyzed by SDS PAGE. The samples were heated to 60° C for 4 min, and resolved on SDS-PAGE using a 4% stacker and 7.5% resolving gel.

### Citrulline colorimetric assay

A colorimetric assay was used for measuring the presence of citrulline using the methodology described by Knipp *et al*. [42]. This assay is based on L-Cit reacting with oximes in strong acids in the presence of Fe^3+^ and high temperature. Briefly, 60 μl of sample was added to the wells of a 96 well plate (Immulon-2HB flat bottom), and 200 μl of COLDER (color developing reagent; 1 vol of solution A and 3 vol of solution B; solution A: 80 mM DAMO and 2.0 mM thiosemicarbazide; solution B: 3 M H_3_PO_4_, 6 M H_2_SO_4_, 2 mM NH_4_Fe(SO_4_)_2_) was added to each well. The plate was then covered with an adhesive sealing film and plate lid, placed in a preheated aluminum pan and covered with a preheated glass plate on top of the microtiter plate to minimize evaporation. Plates were incubated at a 95˚ C for 15 minutes, allowed to cool for 10 minutes, and analyzed using a plate reader to measure absorbance at 530 nm (Spectramax M5, Molecular Devices). L-citrulline was used to produce a standard curve for evaluating the degree of Cit modification of proteins.

### Immunizations and autoimmune arthritis induction

For arthritis induction and T cell proliferation assays, 8 to 10 week old mice were immunized subcutaneously at the base of the tail with 100 μg of peptide or bovine CII emulsified in equal volumes of CFA consisting of 85% heavy paraffin oil (Fisher Scientific), 15% mannide mono-oleate (Sigma), and 4 mg/ml of heat killed mycobacterium (H37Ra, Difco) [43]. For arthritis studies, mice were examined 3 times per week starting at day 18 after immunization and the presence of arthritis, number of affected limbs, and the severity were assessed. Severity of disease was evaluated by visual inspection and assigned a score using a scale of 0 to 4 based on the degree of inflammation as described [43].

### T cell proliferation assays

Ten days after immunization, T cells were recovered from draining lymph nodes of CII-immunized mice. T-cell proliferation assays were performed in 96-well micro-titer plates in a total volume of 0.3 ml containing 4 x 105 lymph node cells and various concentrations of peptide in complete HL-1 medium. Cell cultures were maintained at 37° C in 5% humidified CO2 for 4 days. On day 3 of culture, 1 μCi of [3H]-thymidine was added, and on day 4 the plates were harvested onto filter plates. After the filter plates were dried, scintillation fluid was added and [3H]-thymidine incorporation measured using a Top Count NXT (Perkin Elmer).

### T-cell hybridomas

T-cell hybridomas were established by polyethylene glycol fusion (Boehringer Mannheim) of lymph node cells with BW5147 thymoma cells (TCR α-/β-) [27, 28]. Lymph node cells were obtained from DR1 (DRB1*0101) [24] and DR4 (DRB1*0401) [29] transgenic mice immunized 10 days previously with antigen/CFA. Prior to fusion, lymph node T cells were stimulated antigenic peptide for four days, followed by IL-2 stimulation for three days. Resulting hybridomas were screened for their ability to recognize the peptide presented by DR1 or DR4.

### Antigen presentation assays

Antigen presentation assays were performed in 96-well microtiter plates in a total volume of 0.3 ml containing 5 x 104 antigen presenting cells (APC), 5 x104 T-hybridoma cells, and 10 μg of synthetic peptide (RS Synthesis). Assays were performed in HL-1 medium (Bio-Whittaker) supplemented to 2 mM L-glutamine (Gibco), 50 units/ml penicillin, 50 μg/ml streptomycin (Gibco), and 50 μM β-mercaptoethanol (Gibco). Assay cultures were incubated at 37° C in 5% humidified CO2 for 20 to 24 hours. After this time, culture supernatants were harvested and IL-2 production by the T cell hybridomas was measured in a bioassay using the IL-2 addicted cell line HT-2 [24, 25]. HT-2 cell viability was assessed by cleavage of MTT (Sigma) [30, 31]. IL-2 titers were quantified by the reciprocal of the highest two-fold serial dilution maintaining HT-2 cell viability greater than two-fold over negative control cultures. Results are presented as units of IL-2 per ml as described by Kappler *et al*. [32].

### DR1 binding assay

Soluble DR1 and DR4 were purified from culture supernatants of S2 drosophila cells transfected with DRB1*0101 or DRB1*0401 and DRA1*0101 as described previously [44]. The cytoplasmic and transmembrane portions of these molecules were deleted from the cDNA using PCR, a new stop codon was inserted immediately prior to the transmembrane domain, and the resulting cDNA was cloned into the drosophila expression vector pRmHA-3. S2 cells were transfected with a 10:10:1 ratio of DRB1:DRA1:pUChsNeo using calcium phosphate precipitation. Soluble DR production was induced by 1 mM CuSO4, and 5 days later the culture supernatant was collected and adjusted to 0.05% octyl glucoside (OcG). Soluble DR was purified by passage of the supernatant over an affinity column coupled with the anti-DR antibody L227. The column was washed with 0.05% OcG and 0.15 M NaCl in phosphate buffer (PB), pH 7.5, followed by 0.05% OcG and 0.5M NaCl in PB, pH 7.5. DR was eluted with 100 mM Tris, 0.5M NaCl, pH 11.2, and the fractions were immediately neutralized with acetic acid. Recovered DR protein was concentrated using an EMD Millipore stirred cell with a YM30 disc membrane (Beverly, MA) and quantitated by OD 280 nm absorption and SDS-PAGE prior to use.

For binding assays, a 10 nM solution of purified DR1 or DR4 was incubated for 18 h at 37° C with CII(257–274) peptide (0.5 nM) that had been labeled at the NH2 terminus with biotin as previously described [45]. Various concentrations of peptides were added as competitors to the CII peptide binding. Bound peptides were separated from free peptides by immobilizing the DR molecules on microtiter plates coated with the monoclonal antibody L227 and subsequent washing. The antibody was adhered to the plate by an overnight incubation of a 10 μg/ml solution at 4° C. Bound biotinylated peptides were detected by incubation with streptavidin-europium followed by a chelating enhancement solution. Time resolved fluorescence was quantitated using a microplate fluorometer (FluoroMark, Bio-Rad, Hercules, CA), and data are expressed as relative fluorescence units measured. The 50% inhibitory concentration values (IC50) values were calculated from the binding curves and graphed as 1/IC50 to indicate affinity relative to the control peptide. Each binding assay was performed in duplicate, and data are representative of 3 experiments.

### Cytokine assay

Cytokines produced by WT and Cit-peptide stimulated T cells were measured using a multiplexed bead assay (BioRad) and analyzed using a BioRad MagPix. B6.DR1 mice were immunized with WT or Cit-peptide, and 10 days later LN cells were recovered and re-stimulated with the corresponding WT peptide or Cit-peptide. Supernatants were collected 3 days after stimulation and assayed for expression of IFNγ, TNF*α*, IL-10, IL-17A, and IL-6. Standard curves were used to calculate cytokine concentration. Data are representative of two independent experiments and based on duplicate samples.

## Results

### Effect of citrullination on peptide binding to HLA-DR1 (*01:01) and DR4 (*04:01)

To determine how PTM of self-proteins may influence the development of an autoimmune T cell response, 4 peptide antigens were selected for study based on previous reports that their wild type (WT) or citrullinated (Cit) form may be involved in autoimmune T cell responses in RA [24, 34, 38, 46, 47]. As shown in Table 1, these four antigenic peptides, derived from aggrecan (Agg), vimentin (Vim), type II collagen (CII), and fibrinogen (Fib), all have Arg residues at positions that are likely involved in either peptide binding to HLA-DR1 (*01:01) or DR4 (*04:01), or interaction with the T cell receptor (TCR) when presented by either of these HLA-DR alleles [21, 35]. All of these peptide sequences are native and based on human proteins, and the Agg and Vim peptides differ from previously published data on this basis [20, 36].

**Fig 1:**
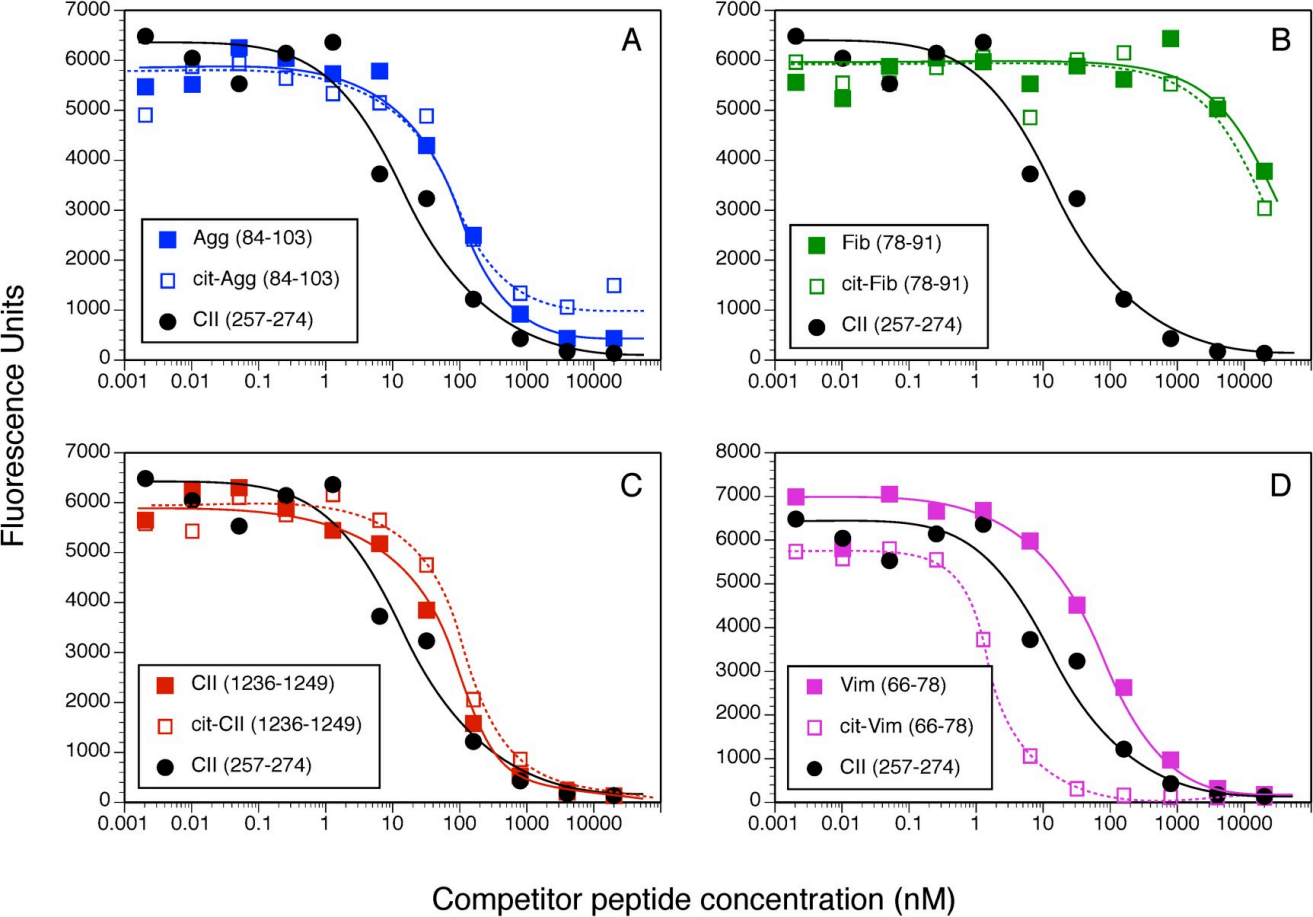
Measurement of peptide binding to DRB1*01:01. 10 nM of soluble DR1 was incubated for 18 h at 37° C with 0.5 nM of biotinylated CII(257–274) peptide and various concentrations of WT and Cit-peptides as competitors. Bound peptides were separated from free peptides by immobilizing the DR molecules with an antibody on microtiter plates. Bound biotinylated peptide was detected by incubation with streptavidin-europium. Time resolved fluorescence was quantitated and data are expressed as relative fluorescence units measured. Each binding assay was performed in duplicate, and data are representative of 3 experiments.

**Fig 2:**
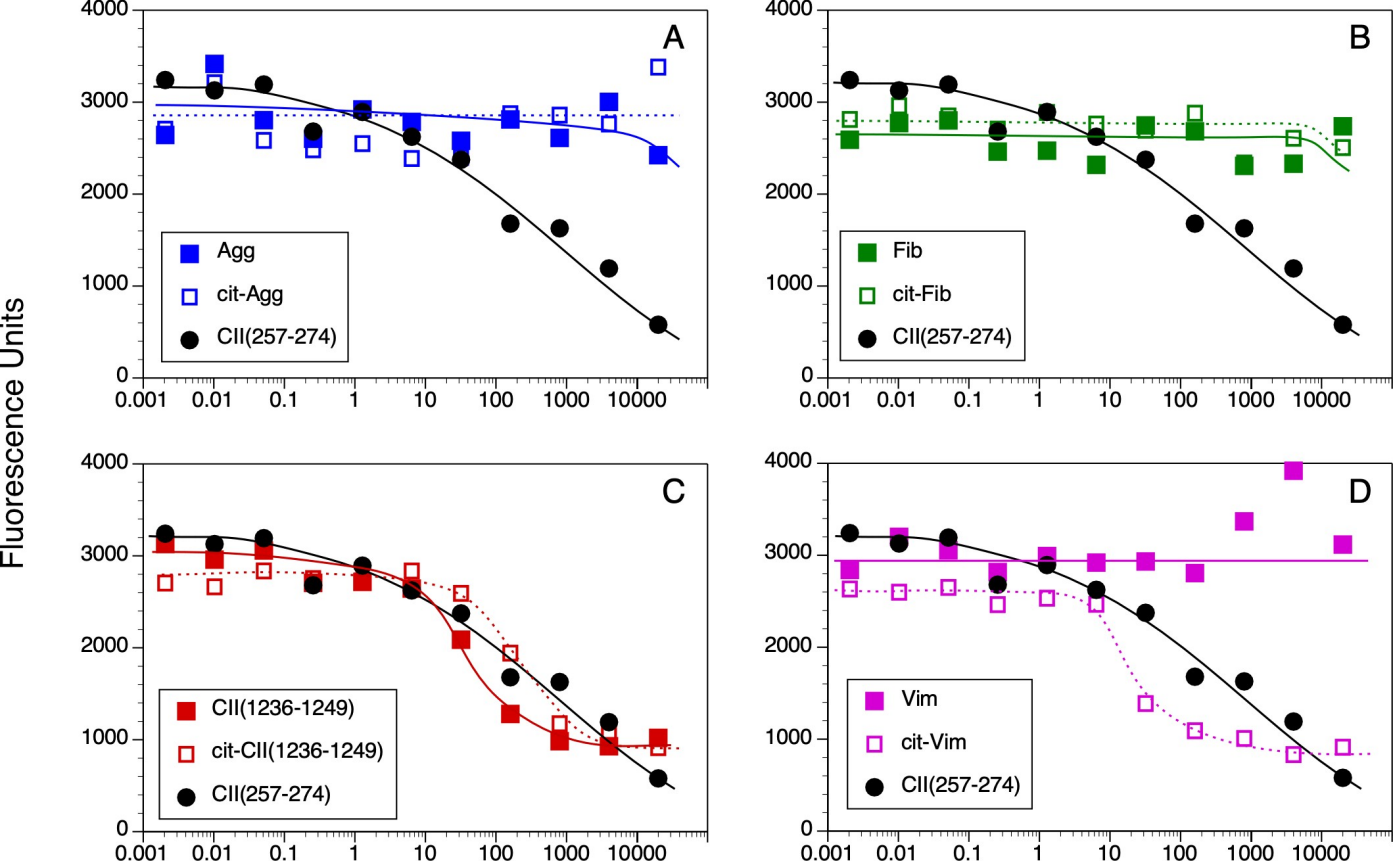
Measurement of peptide binding to DRB1*04:01. 10 nM of soluble DR4 was incubated for 18 h at 37° C with 0.5 nM of biotinylated CII(257–274) peptide and various concentrations of WT and Cit-peptides as competitors. Bound peptides were separated from free peptides by immobilizing the DR molecules with an antibody on microtiter plates. Bound biotinylated peptide was detected by incubation with streptavidin-europium. Time resolved fluorescence was quantitated and data are expressed as relative fluorescence units measured. Each binding assay was performed in duplicate, and data are representative of 3 experiments.

**Fig 3:**
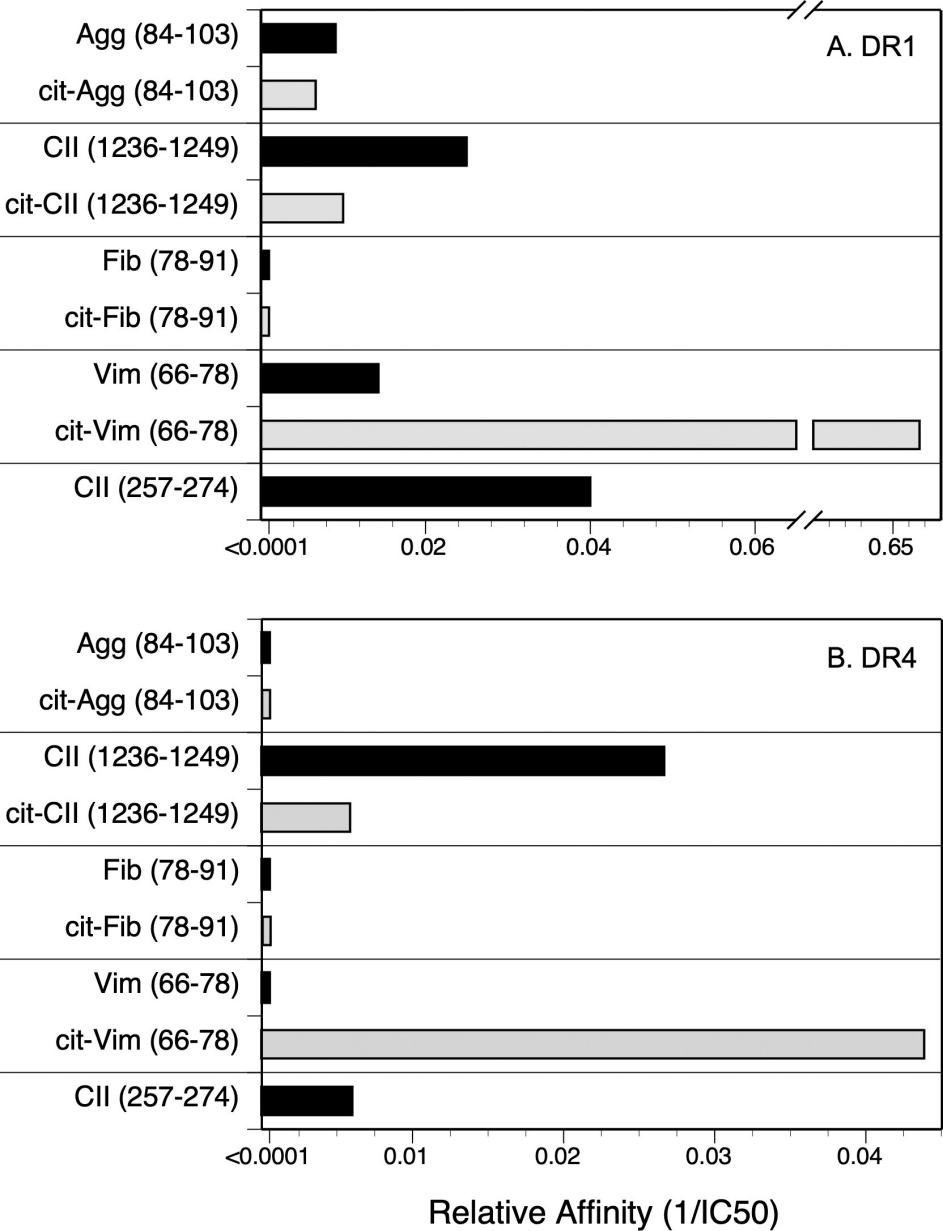
Relative affinity of WT and Cit-peptide binding to DRB1*01:01 and DRB1*04:01. The 50% inhibitory concentration values (IC_50_) values were calculated from the binding curves in Figs 1 and 2, and plotted as 1/IC_50_ to indicate relative affinity. Panel A, DR1 binding; panel B, DR4 binding.

Competitive binding assays were used to evaluate the contribution of Cit substitution for Arg in each of these peptides using HLA-DRB1*01:01 (DR1, Fig 1) and DRB1*0401 (DR4, Fig 2) and IC50 were calculated to evaluate the relative affinities (Fig 3). The CII(257–274) peptide was used as a comparison control as this peptide binds to DR1 and DR4 and induces an autoimmune T cell response in animals expressing these DR alleles [39, 40, 45, 48]. All of the WT versions of the peptides in Table 1 bound to DR1, albeit at varying affinities (Fig 3A). The CII(1236–1249), Agg, and Vim peptides bind to DR1 with a lower affinity in comparison to the competitive control peptide, CII(257–274) [19, 45] (IC50 82.9 nM, 110.1 nM, and 76.1 nM versus 5.9 nM, respectively), and the Fib peptide binds poorly (IC50 >4000 nM) (Fig 1). For 3 of these peptides, Agg Fib, and CII, the substitution of Arg with Cit had little to no effect on their affinity for DR1 (Figs 1A–1C and 3A). Indeed, the affinity of the Cit-Agg and Cit-CII was not changed compared to their WT counterpart (IC50 82.6 nM versus 110.1nM for Agg WT vs cit peptides, and IC50 121.1 nM and 82.9 nM for the CII WT vs Cit peptides), and citrullination of the Fib peptide did not translate into any beneficial effect on the binding of the Fib peptide (Figs 1 and 3A). In contrast, Cit substitution for the Arg at position 71 in the Vim peptide significantly increased the affinity of the peptide by nearly 100-fold (IC50 1.1 nM) in comparison to the WT Vim peptide (IC50 76.1 nM) (Figs 2D and 3A).

The WT and Cit versions of these peptides bound to DR4 with significantly less affinity in comparison to DR1 binding (Figs 2 and 3B). Binding of the WT Agg and Fib peptides to DR4 was essentially undetectable even at a 40,000:1 nM ratio of competitor:indicator peptide (Fig 2A, 2B, and 2D), and substitution of the Arg residues with Cit within the DR4 binding motif in these peptides did not improve their binding affinity. The WT CII(1236–1249) peptide binds to DR4 with a slightly higher affinity than the CII(257–274) peptide (IC50 37.4 nM and 166.1 nM, respectively; Figs 2C and 3B), however substitution of the Arg with Cit decreased its affinity for binding to DR4 (IC50 170.3 nM). Similar to the DR1 binding data, citrullination of the Arg in the P4 position of the Vim peptide significantly increased its affinity for DR4 (Figs 2D and 3B). Yet the presence of Cit at P4 is not a universal enhancer for binding affinity for DR1 or DR4, as Cit in the P4 position of the Fib peptide and the CII(1236–1249) peptide for DR1 did not generate a peptide with a higher binding affinity for these HLA-DR alleles (Fig 3).

### HLA-DR1 and DR4-restricted T cell responses to citrullinated antigens

**Fig 4:**
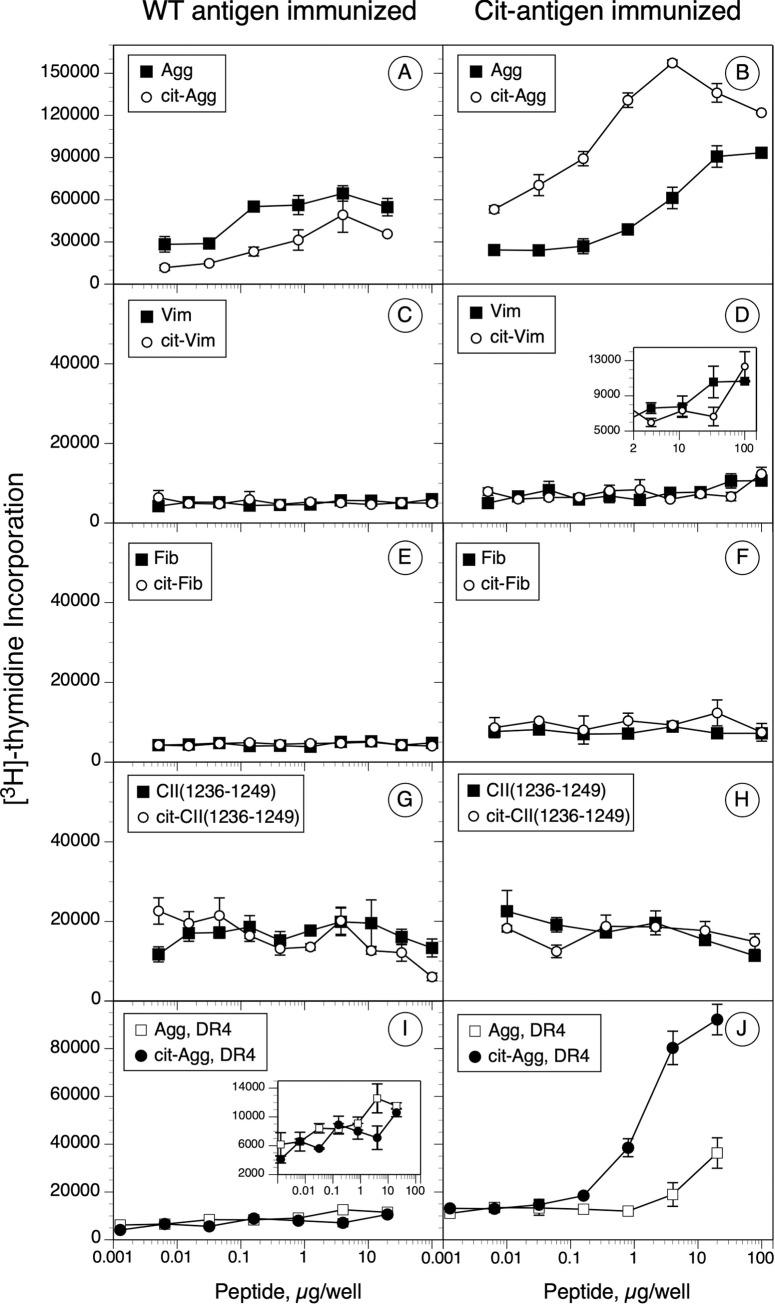
Immunogenicity and antigenicity of WT and Cit-peptides in DRB1*01:01 and DRB1*04:01 humanized mice. B6.DR1 and B6.DR4 mice were immunized with the WT and Cit-peptides, and 10 days later, T cells from draining lymph nodes were cultured in the presence of various concentrations of peptides for 4 days. On day 3, [^3^H]-thymidine was added to the cultures and cells were harvested the following day. Data in the panels on the left were derived from WT peptide immunized mice; data on the right were from the corresponding Cit-peptide immunizations. Panels A thru H are from B6.DR1 mouse immunizations; panels I and J are from B6.DR4 immunized mice. Controls were unstimulated cells, and data shown are Δ cpm (experimental minus control). Error bars indicate standard deviation of triplicates, and data are representative of two independent experiments.

To determine the relationship between peptide binding affinity, citrullination, and the stimulation of a T cell response, DR1 and DR4 mice were immunized with the WT or Cit-peptides from Table 1 and T cell proliferative responses and cytokine production were evaluated. Through these experiments we sought to determine 1) if peptide affinity would predict a T cell response; 2) if Cit-peptides were more immunogenic than the WT forms; and 3) if T cells primed by WT or Cit peptides cross recognized the corresponding antigenic peptides. As shown in Fig 4, the T cell responses to this panel of peptides varied widely. Two of the antigenic peptides, Agg and Cit-Agg, stimulated a strong T cell proliferative response, while a potentially weak response was observed with Cit-Vim. Immunization of DR1 mice with the Agg peptide stimulated a moderate response that weakly but definitively cross recognized the Cit-Agg peptide (Fig 4A). In comparison, immunization with Cit-Agg generated a strong T cell response that cross recognized the WT Agg peptide, although the magnitude of the cross-reactive response was smaller than the Cit-Agg stimulation (Fig 4B). Similar results were obtained for the DR4 mouse immunizations with the Agg peptides (Fig 4I and 4J). While immunization of the DR1 mice with the Vim antigen did not generate a T cell response (Fig 4C), immunization with the Cit-Vim appeared to induce a weak response (Fig 4D). Immunization with either form of the Fib peptide or the CII(1236–1249) peptide failed to generate a detectable T cell proliferative response (Fig 4E–4H). Collectively, these data indicate a disconnect between peptide affinity and induction of a T cell response. Agg and Cit-Agg bind with low affinity to DR1 (IC50 110.1 & 82.6 nM, respectively, Fig 3A) and binding to DR4 was nearly undetectable (IC50 >5000 nM each, Fig 3B), yet these peptides stimulated the strongest T cell responses. In contrast Cit-Vim binds with high affinity to DR1 (IC50 1.1 nM) yet is very poor at stimulating a DR1-restricted T cell response. Additionally, while 2 of the Cit-peptides were capable of stimulating a T cell response (Cit-Agg and Cit-Vim), citrullination of the other peptide antigens had no effect on their T cell immunogenicity.

**Fig 5:**
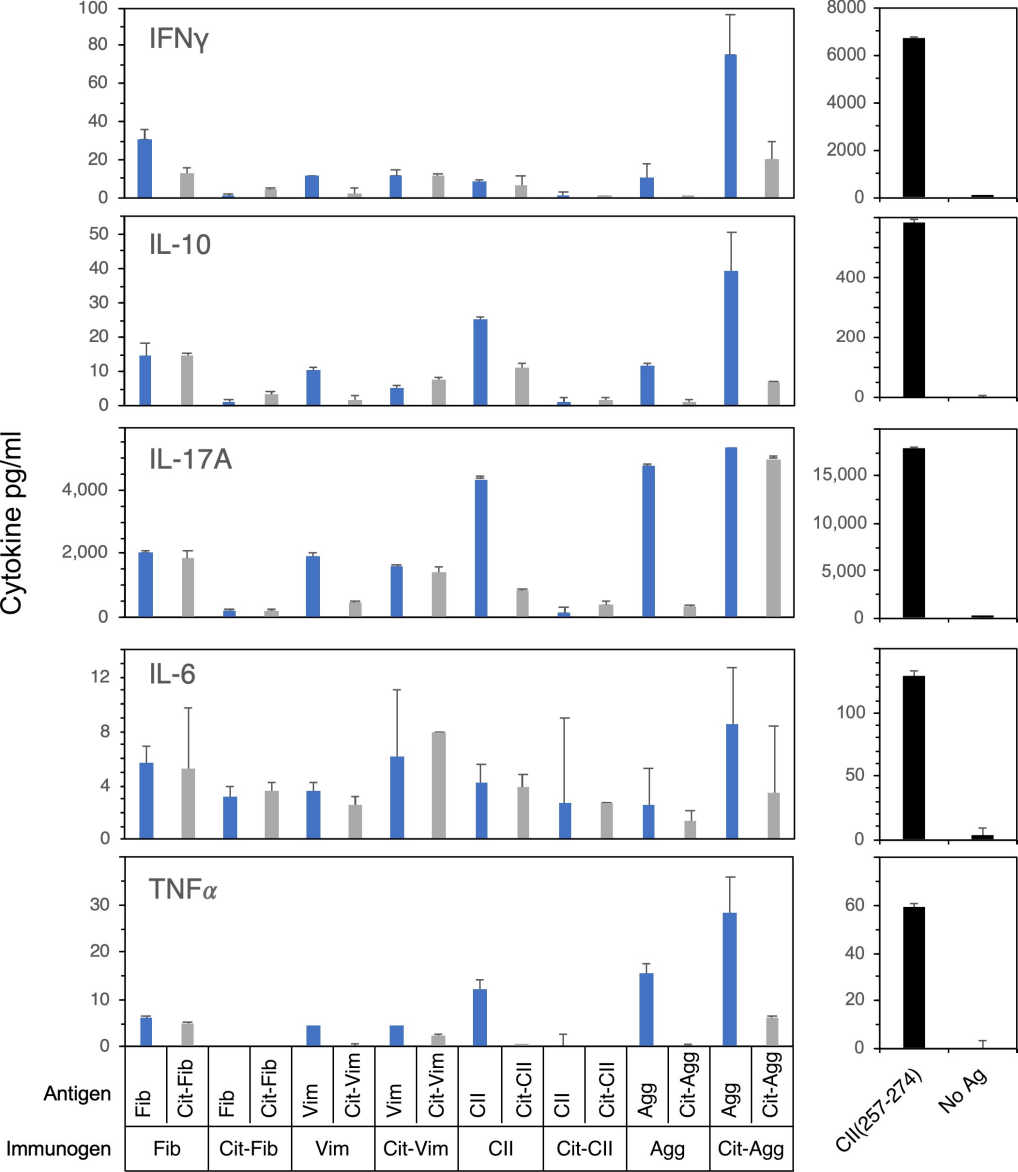
Cytokine production by T cells stimulated with WT or Cit-peptides. Supernatants from T cell proliferation assays in Fig 4 were collected 3 days after antigen stimulation and analyzed for the expression of proinflammatory cytokines. Cytokines produced were measured using a multiplexed bead assay and analyzed using a BioRad MagPix. Standard curves were used to calculate cytokine concentration. The cytokines produced by CII immunized T cells stimulated with the immunodominant epitope, CII(257–274), that drives autoimmune arthritis in the B6.DR1 model are shown for comparison (right panels). Data are representative of two independent experiments and based on duplicate samples.

As shown in Fig 5, cytokine production by these T cells following antigen stimulation was assessed as a qualitative and quantitative measurement of T cell effector function. In comparison to immunization and stimulation with the CII(257–274), the immunodominant determinant for eliciting autoimmune arthritis in this model, none of the WT or Cit modified antigens induced significant levels of IFNγ, TNF*α*, or IL-6, either with stimulation with immunogen or cross recognition of the WT or Cit form of the antigenic peptide (Fig 5). Despite the inability of several of the antigens to stimulate a T cell proliferative response, nearly all of the WT antigens stimulated the production of significant quantities of IL-17A. While the antigens that stimulated the strongest T cell proliferative response also stimulated the highest levels of IL-17A (Agg and Cit-Agg), the WT CII peptide also stimulated high levels of IL-17A production without stimulating a detectable proliferative response, but this stimulation did not crossover to the Cit-CII peptide. Overall, aggrecan and Cit-aggrecan induced the strongest production of proinflammatory cytokines. Collectively, the T cell response to aggrecan is the most interesting as it supports the hypothesis that an induced immune response to a posttranslationally modified antigen can cross react with unmodified, native protein and potentially initiate or perpetuate an autoimmune T cell response.

### T cell receptor cross-recognition of the WT and Cit peptides

**Fig 6:**
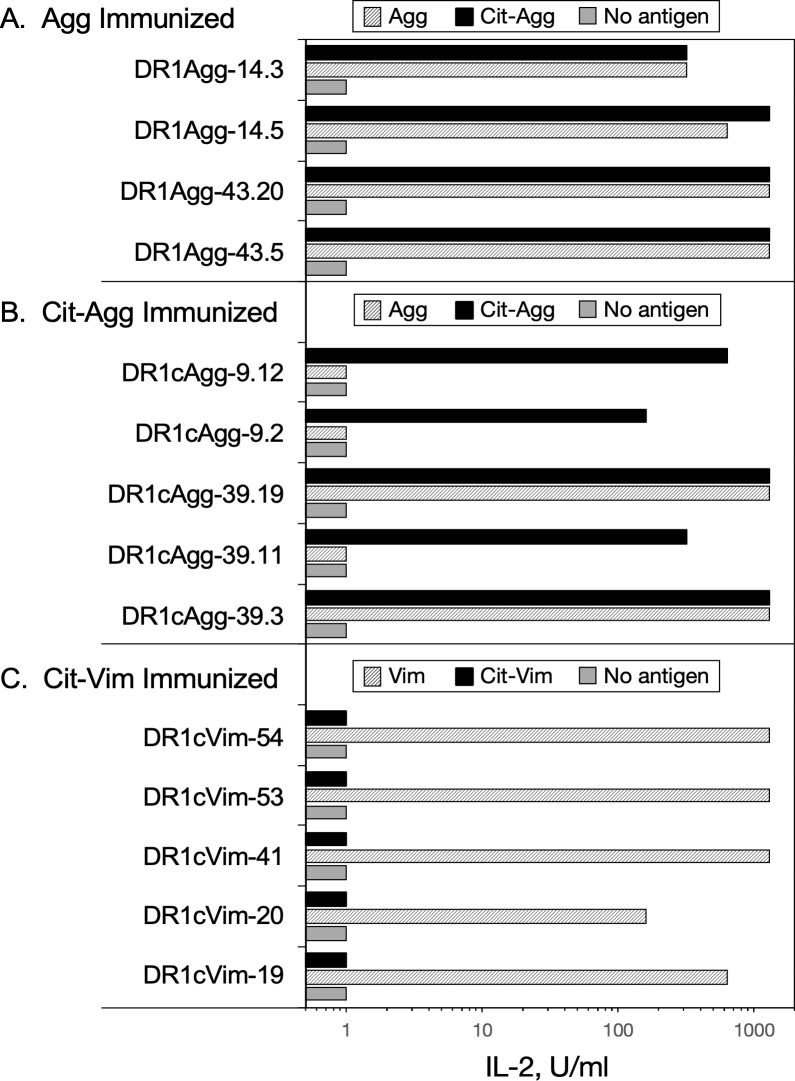
T cell receptor cross recognition of WT and Cit-peptides. Clonal populations of T cells were tested for their ability to recognize WT or Cit-peptides in antigen presentation assays as described in Materials and Methods. T cells from Agg, Cit-Agg, and Cit-Vim immunized mice were used to produce T-cell hybridomas, and multiple clones from each were tested for their ability to recognize the immunogen and the corresponding WT or Cit-peptide. Data indicate that cross recognition of WT and Cit-peptides can occur at the clonal level, and that Cit-peptides can be novel antigens for T cell responses. Data shown are representative responses from multiple clones tested from each immune T cell population.

While the T cell proliferation data indicate the existence of cross-reactive responses between WT and the corresponding Cit-peptides, these data do not differentiate between TCR cross recognition of the corresponding WT or Cit-antigen, or if the inflammatory milieu of the immune response generated the Cit-antigens and the data instead reflect epitope spreading. To address this question, clonal populations of T cell hybridomas were produced from DR1 mice immunized with Agg, Cit-Agg, or Cit-Vim, and their ability to cross-recognize their corresponding WT or Cit-peptide was determined using antigen presentation assays (Fig 6). Immunization with Vim peptide failed to generate any Vim-specific T cell hybridoma clones. All of the Agg-specific T cell clones generated from the Agg immunized mice clearly recognized the Cit-Agg peptide, indicating that the TCRs expressed by these clones do not differentiate between the WT and Cit versions of the same peptide. In contrast, the TCR expressed by the Cit-Agg-specific T cells fall into two categories–those that recognize only the Cit-Agg peptide and those that recognize both Cit and WT Agg. Given that the Cit addition to Agg does not significantly increase its affinity for DR1, collectively these data indicate that the Cit residue is likely being contacted by the TCR and that the stimulation of T cells with Cit-Agg can drive epitope spreading by generating clones capable of recognizing the WT form of the Agg peptide. However, based on the data from the Cit-Vim T cell clones, this is not a universal rule for citrullinated antigens. Despite the weak T cell response from immunization of DR1 mice with the Cit-Vim peptide, DR1-restricted T cells specific for Cit-Vim were generated and all of these clones recognized only the Cit-Vim. Given that the Cit in Vim is in the P4 position, these data imply that the binding of the Cit-Vim peptide generates a DR1-Cit-Vim complex that differs stereotypically from the DR1-Vim complex as perceived by the TCR.

### Citrullination of the autoantigen and induction of autoimmunity

**Fig 7:**
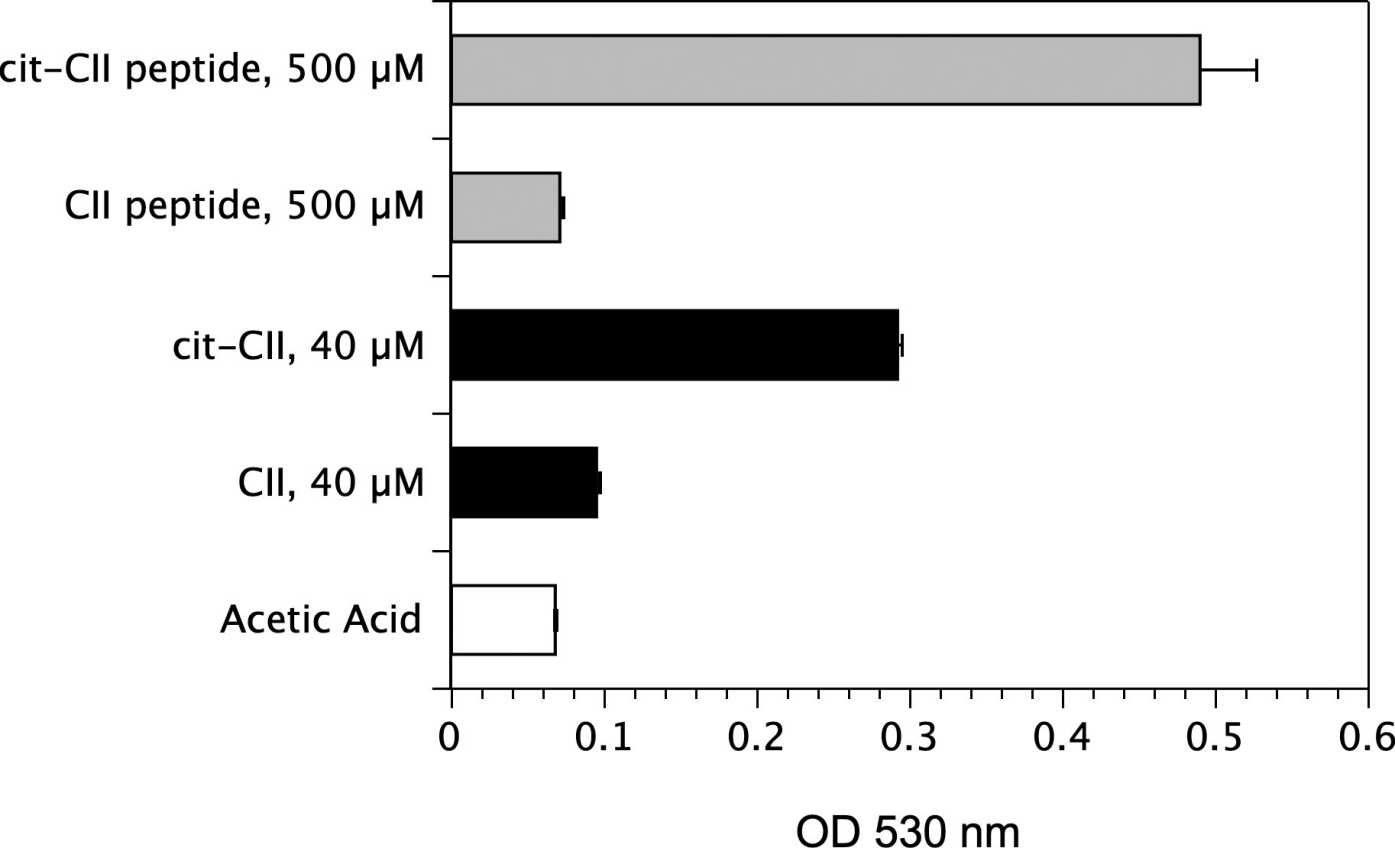
Measurement of PAD4-meidated citrullination of CII. Native bovine CII was citrullinated using PAD4, and the degree of citrullination achieved was measured using a colorimetric assay and absorbance at 530 nm. Cit-CII peptide was produced synthetically and used for comparison. All samples were dissolved in 10 mM acetic, and this reagent was used as the negative control. Data represent triplicate measurements, and error bars represent standard deviation.

**Fig 8:**
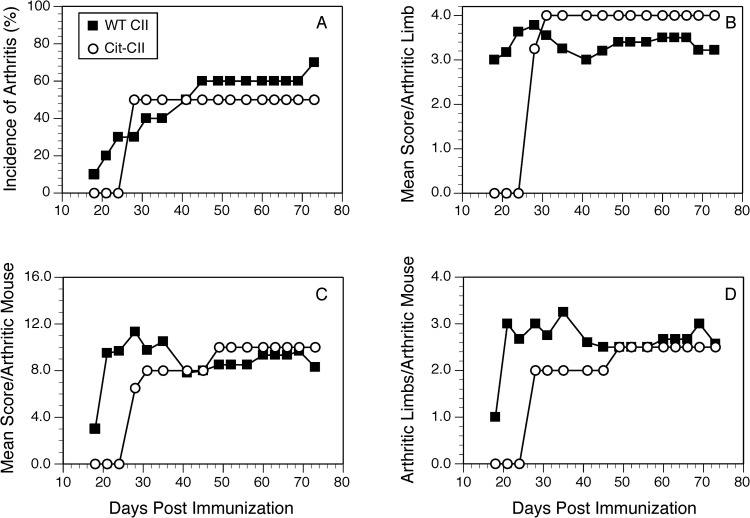
Induction of autoimmune arthritis in HLA-DR1 mice with WT CII and Cit-CII. B6.DR1 mice were immunized with native CII or Cit-CII and the incidence (panel A) and severity (panels B, C, and D) of arthritis were monitored. Disease severity was evaluated on the basis of arthritic animals only, and was assessed using a scale of 0 to 4 as described in Materials and Methods. No significant differences were observed in the incidence or severity of disease between the two groups. Data are representative of two independent experiments.

Given that our T cell response data indicated that citrullination of some proteins can enhance their immunogenicity and initiate epitope spreading, we tested the arthritogenicity of citrullinated CII (Cit-CII) in comparison with WT CII using an HLA-DRB*01:01 transgenic mouse model of autoimmune arthritis [39]. To generate Cit-CII, native bovine CII was treated with PAD4 to convert Arg to Cit [23, 41], and the efficacy of the citrullination was measured using a colorimetric assay based on the Cit reacting with oximes in the presence of Fe^3+^ [42] (Fig 7). These data clearly demonstrate the conversion of Arg to Cit, although based on the comparison of molar quantities with the citrullinated peptide, it appears that not all Arg in the native CII were converted. Immunization of the DR1 Tg mice with the Cit-CII induced autoimmune arthritis, however there was no evidence that the disease was more severe than immunization with WT CII (Fig 8). Incidence of disease (Fig 8A), onset, and severity (Fig 8B–8D) were similar between these two groups. Although it appeared that the mean severity score per arthritic limb was higher in the Cit-CII group (Fig 8B), this difference was not statistically significant. While the immunodominant core determinant of CII, residues 263–270, does not contain any Arg residues and therefore cannot be affected by citrullination, these data indicate that citrullination of other Arg residues within CII does not appear to generate novel epitopes or generate epitope spreading events that enhance the severity of the autoimmune arthritis.

## Discussion

The results of these studies demonstrate that citrullination of proteins can have a wide range of effects on T cell immune responses mediates by SE+ MHC-II alleles. Using a panel of antigenic peptides proposed to be targeted by T cells in RA, we found that substitution of Arg with Cit does not universally enhance their bind affinity to DRB1*01:01 or DRB1*04:01, even when the Cit was in the P4 anchor position. Additionally, peptide binding affinity did not predict the generation of a T cell immune response. While the majority of the peptides studied had binding affinities similar to a control peptide known to induce a T cell response [39, 45], only 3 of the 8 peptides studied (Agg, Cit-Agg, and Cit-Vim) stimulated a measurable T cell proliferative response. However, when these T cell responses occurred, their specificity provided clear evidence of neoepitope generation dependent on the presence of the Cit residue, as well as evidence that T cell cross recognition of WT peptides can occur as a result of an immune response initiated by the citrullinated antigen. In all, these data provide evidence supporting the potential role of Cit-proteins in generating an autoimmune T cell response.

The purpose of these studies was to test the hypothesis that immunization with PTM proteins or peptides will stimulate enhanced CD4+ T cell responses that are comprised of both cross reactive as well as T cells specific for the novel citrullinated antigen. Given that expression of PAD enzymes is induced at sites of inflammation [49], the generation of novel PTM proteins at these sites represents a potential source of autoimmune T cell generation. While the MHC-II alleles expressing the SE appear to have some propensity to accommodate a citrulline at the P4 position for peptide binding [20, 21], our data and others [35] indicate that this is not a universal phenomenon of citrullinated antigenic peptides. Of the 4 antigenic peptides we tested, only 1 bound to DR1 with a higher affinity, and despite this higher affinity, it stimulated only a weak T cell response. While collectively these data do not negate the potential role of citrullinated peptides as initiators or propagators of an autoimmune T cell response, they do demonstrate the complex relationship of citrullination of self-protein antigens, presentation by MHC-II alleles, and the ability of these Cit-peptide:DR1 complexes to stimulate a T cell response. An interesting aspect of our data is that many of these putative RA autoantigenic peptides bind to DR1 and DR4 with affinities similar to the CII(257–274) peptide that is the immunodominant determinant in the DR1 humanized mouse model of autoimmune arthritis and stimulates a strong T cell response. Yet despite most having similar or in some cases higher affinity (Cit-vimentin), the T cell proliferative responses to most of the antigens we tested were undetectable. Only Cit-aggrecan stimulated a significant T cell response and the Cit-Vim response was barely detectable. One obvious explanation is that thymic selection has deleted the T cells that had the potential of recognizing these antigens, or T cells specific for these antigens have been tolerized. Why that would differ for the CII(257–274) determinant of type II collagen is unclear, especially given that another peptide from CII, CII(1236–1249), binds with similar affinity and also does not induce a T cell response.

For two of the antigenic peptides we tested, Vim and Agg, our MHC-II binding data differ from that published by Hill *et al*. [36] and Scally *et al*. [20], respectively. For both peptides, we used the native sequence of the human protein while both previously published studies used peptides with amino acid substitutions to improve the binding of the peptide in order to investigate the contribution of Cit at the P4 position to HLA-DR binding affinity. Without these substitutions, we found that the Agg and Vim WT peptides bind at a significantly lower affinity than reported in these previous studies. The goal of our studies was to investigate how citrullination of a protein affected the T cell recognition and whether T cells stimulated by the Cit-peptide would cross react with the native protein. The T cell responses observed to the Agg peptides and Cit-Vim provided evidence of both epitope spreading and the formation of neoepitopes. Using clonal populations derived from the T cell proliferative responses, clones from the Agg immunized mice cross recognized the Cit-Agg in every instance tested, indicating the TCR recognition of these antigens was not affected by the presence of the Cit. In contrast, T cells clones from the Cit-Agg immunized mice were evenly divided between TCR that only recognized the Cit-Agg and those that also cross reacted with the WT Agg peptide. Immunization with Cit-Vim generated T cells that only recognized the Cit-Vim neo-epitope; no cross recognition of the WT Vim peptide was observed. Collectively, these data demonstrate that, in addition to peptide binding, citrullination of a protein can generate a Cit-specific T cell response without the citrulline enhancing the affinity of peptide binding to MHC-II. Given that most of the Cit substitutions were in the P4 position, these data imply that the Cit residue alters the conformation of the DR1:peptide complex in comparison the WT peptide, thus representing a different molecular interface for the TCR. We have observed similar conformational changes induced in HLA-DR1 and DR4 when bound to the same antigenic peptide [19]. In all, these data support the concept that citrullination of proteins can also lead to the development of T cell responses to a novel antigen that then have the potential for cross reactive autoimmune recognition of the WT self-protein.

The cytokine production and proliferation profile by T cells from WT and Cit-antigen immunized mice was unexpected. First, IL-17A was produced by T cells from most of the antigen stimulations, regardless of whether the antigen induced a measurable T-cell proliferative response or not (Table 2). Second, immunization and restimulation with CII (1236–1249) failed to induce a detectable T cell proliferative response, but did result in cytokine production. Of all the antigens studied, the production of proinflammatory cytokines was most striking with the stimulation of Cit-Agg primed T cells with WT aggrecan, implying that cross recognition can play a role in generating pathogenic T cell responses. Whether the lack of a detectable proliferative response is due to a regulatory mechanism induced by the antigen or indicative of a very low precursor frequency of T cells that recognize these antigens is unclear. The combination of these observations with the DR1 binding data presented here and from earlier studies raises several questions. All of the antigens, both wild type and citrullinated forms, clearly bind to DR1, and for the most part, bind with an affinity that is at least as strong as the immunodominant peptide CII(257–274) that drives the development of autoimmune arthritis in the DR1 mouse model of RA. How this relationship between affinity and T cell stimulation leads to the development of an autoimmune T cell response is unclear. Given that peptide binding affinity does not appear to be the sole factor, loss of tolerance or T cell regulation are possible explanations as to how the autoimmune T cell response is initiated. If tolerance prevents the development of a T cell response to the majority of the antigens studied, then both the mechanism by which the CII(257–274) determinant escapes tolerance and the means by which tolerance to the other antigens is maintained should be further investigated. It is unclear if citrullination of proteins occurs in the thymus under the control of AIRE or Fezf2 [50]. The absence of this PTM in the thymus would allow the Cit-specific T cells to escape to the periphery with the potential for future autoimmune T cell responses. A second mechanism that could be involved in preventing the T cell response to these antigens is the generation of Treg cells by the PTM immunogens. Given the IL-10 production in several of the studies described here, and that some populations of Treg cells produce IL-10 [51–53], we analyzed T cell populations post- immunization and restimulation *in vitro* with WT and Cit-peptide to see if the Treg population is expanded in any of these instances. No evidence of Treg expansion was observed (unpublished observation, S. Becart and E. Rosloniec).

**Table 2:** Summary of DR binding, T cell proliferation, and cytokine production as stimulated by WT or Cit antigenic peptide.

		T cell Proliferation Stimulation Index^a^		Cytokine WT/Cit Ratio^b^				
Immunogen	IC50 for DR1 (nM)	WT Ag	Cit Ag	IFNγ	IL-10	IL-17A	IL-6	TNFα
Fib	>4500^c^	0.9	0.8	2.5	1.0	1.4	1.1	1.3
cit-Fib	>4500	0.9	1.0	0.2	0.3	1.0	0.9	–
Vim	76.1	1.0	0.8	5.5	6.6	3.9	1.4	> 4
cit-Vim	1.1	1.5	1.7	1.1	0.7	1.1	0.8	1.9
CII	82.9	1.0	1.0	1.3	2.3	4.4	1.1	59.5
cit-CII	121.1	1.1	0.9	0.6	0.5	0.4	1.0	–
Agg	110.1	1.6	1.4	49.4	9.9	13.0	1.8	> 15
cit-Agg	82.6	2.7	6.9	3.7	5.4	14.4	2.5	4.7

^a^Stimulation indices were calculated from the T cell proliferation data in Fig 4.

^b^Cytokine ratios were calculated from the data in Fig 5. TNFα cytokine ratio values of “>” are the result of no TNFα produced by Cit-Ag stimulation, and “–” values the result of no cytokine detected from the Cit-Ag or both antigens.

^c^Colors indicate strength of binding (IC50, green strongest to red for the weakest), strength of T cell proliferation (Proliferation Index, green indicating strongest and red weakest), and cytokine ratio (WT/Cit peptide ratio) with green indicating cytokine produced by Cit stimulation was equal or stronger than the stimulation with WT, and red indicating a stronger WT stimulation than Cit.

While other studies with Cit-CII indicated some enhanced ability to induce autoimmune arthritis in other mouse [54, 55], and rat models [23], Cit-CII was not a more potent autoantigen in our HLA-DR1 humanized mouse model of RA. While the DR1 and DR4 immunodominant determinant of CII is unaffected by citrullination (it does not contain Arg), these results indicate that citrullination of other Arg residues in CII does not appear to generate novel T cell determinants that exacerbate the pathogenesis in this HLA-DR mouse model of RA. Maintaining the native structural conformation of CII for immunization is important for high penetrance of disease in this model. We cannot rule out that some denaturation of CII occurred during the citrullination protocol, and that may have reduced the capacity of the Cit-CII to induce a pathogenic autoimmune response. That said, the disease incidence was unchanged in comparison to the control sham-treated CII, and the overall incidence of disease for both groups was slightly less than the 80 to 100% routinely observed [56]. Additionally, we cannot confirm that all Arg were converted to Cit by PAD4, and there is some substrate specificity that regulates the efficiency of PAD4 citrullination [57]. Using a colorometric assay, measurement of the efficacy of the PAD4 enzyme in citrullinating CII indicated residues were converted, but the data also indicated that it was unlikely that all Arg in all CII molecules were affected. In preliminary studies, mass spec sequencing of citrullinated and WT CII has revealed that WT CII recovered from fetal bovine cartilage also contains citrullinated residues (unpublished observation, S. Becart & E. Rosloniec), suggesting that the arthritogenicity of native CII in our model may be dependent on PTM already present.

Altogether, these data demonstrate that the generation of an autoimmune response to citrullinated proteins and peptides is a complex process. Several hypotheses have been proposed including autoimmune responses to the PAD4 protein in which the PAD4 acts as carrier and the citrullinated proteins act as a hapten [58, 59], as well as a potential role of non-SE MHC-II alleles participating in the presentation of citrullinate peptides [60]. Additionally, citrullination is only one of many PTMs that can occur to proteins, raising the possibility that autoimmune T cell responses could be targeting multiple neo-antigens that have been generated at the site of inflammation. Through the data described here and from others, it is clear that citrullination can generate novel antigenic peptides that now bind to MHC-II, and that some of these peptide:MCH-II complexes can stimulate a T cell response that is both dependent on the presence of the PTM and produces pro-inflammatory cytokines. Perhaps the most surprising aspect of these data is the independent nature of each of these biological events. PTM peptide affinity for HLA-DR did not predict immunogenicity for T cells, and the strength of the T cell proliferative response did not predict the cytokine response. How all these biological factors function collectively in the generation of a pathogenic autoimmune T cell response remains to be further elucidated.
